# *N*-(3,4-Difluoro­phen­yl)-2-(3,4-dimethoxy­phen­yl)acetamide

**DOI:** 10.1107/S1600536808003590

**Published:** 2008-02-06

**Authors:** Won Ki Hong, You-Soon Lee, Byung Hee Han, Sung Kwon Kang, Chang Keun Sung

**Affiliations:** a Department of Chemistry, Chungnam National University, Daejeon 305-764, Republic of Korea; b Department of Food Science and Technology, Chungnam National University, Daejeon 305-764, Republic of Korea

## Abstract

In the title amide, C_16_H_15_F_2_NO_3_, the dihedral angle between the benzene rings is 53.7 (1)°. Mol­ecules are linked in the crystal structure by an inter­molecular N—H⋯O hydrogen bond involving N—H and C=O functionalities of the amide group. A one-dimensional network is thus formed along the [001] direction. No significant inter­chain contacts are observed.

## Related literature

For general background, see: Maeda *et al.* (1991 ▶); Dawley *et al.* (1993 ▶); Nerya *et al.* (2003 ▶); Lee *et al.* (2007 ▶); Ha *et al.* (2007 ▶); Hong *et al.* (2008 ▶); Yan *et al.* (2007 ▶).
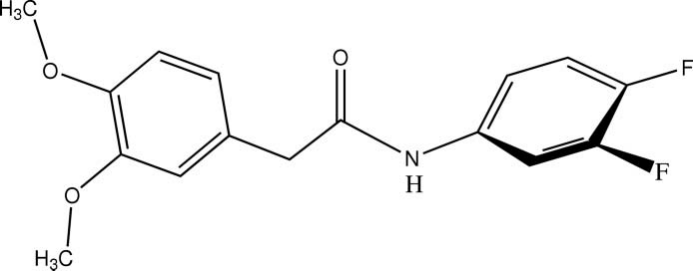


## Experimental

### 

#### Crystal data

C_16_H_15_F_2_NO_3_
*M* *_r_* = 307.29
Monoclinic, 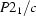

*a* = 8.6440 (11) Å
*b* = 18.867 (6) Å
*c* = 9.4827 (13) Å
β = 111.019 (11)°
*V* = 1443.6 (5) Å^3^
*Z* = 4
Mo *K*α radiation
μ = 0.12 mm^−1^
*T* = 295 (2) K
0.26 × 0.26 × 0.23 mm

#### Data collection

Enraf–Nonius CAD-4 diffractometer
Absorption correction: none
2855 measured reflections
2689 independent reflections
1089 reflections with *I* > 2σ(*I*)
*R*_int_ = 0.050
3 standard reflections every 400 reflections intensity decay: 3%

#### Refinement

*R*[*F* ^2^ > 2σ(*F* ^2^)] = 0.076
*wR*(*F* ^2^) = 0.161
*S* = 0.99
2689 reflections
203 parameters
H atoms treated by a mixture of independent and constrained refinement
Δρ_max_ = 0.19 e Å^−3^
Δρ_min_ = −0.19 e Å^−3^

### 

Data collection: *CAD-4 EXPRESS* (Enraf–Nonius, 1994 ▶); cell refinement: *CAD-4 EXPRESS*; data reduction: *XCAD4* (Harms & Wocadlo, 1995 ▶); program(s) used to solve structure: *SHELXS97* (Sheldrick, 2008 ▶); program(s) used to refine structure: *SHELXL97* (Sheldrick, 2008 ▶); molecular graphics: *ORTEP-3 for Windows* (Farrugia, 1997 ▶); software used to prepare material for publication: *WinGX* (Farrugia, 1999 ▶).

## Supplementary Material

Crystal structure: contains datablocks global, I. DOI: 10.1107/S1600536808003590/bh2160sup1.cif

Structure factors: contains datablocks I. DOI: 10.1107/S1600536808003590/bh2160Isup2.hkl

Additional supplementary materials:  crystallographic information; 3D view; checkCIF report

## Figures and Tables

**Table 1:** Hydrogen-bond geometry (Å, °)

*D*—H⋯*A*	*D*—H	H⋯*A*	*D*⋯*A*	*D*—H⋯*A*
N7—H7⋯O9^i^	0.89 (5)	1.98 (5)	2.846 (5)	163 (5)

Symmetry code: (i) 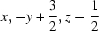.

**Fig. 1.**
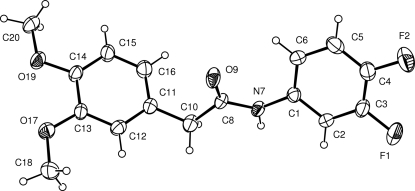
The molecular structure of the title compound, showing the atom-numbering scheme and 30% probability ellipsoids.

## supplementary crystallographic information

### Comment

Tyrosinase is the key enzyme (Ha *et al.*, 2007) that converts tyrosine to melanin, and its inhibitors are the target molecules to develop and research anti-pigmentation agents for application to skin. The melanin formation is also accelerated by exposure under sunlight, especially U.V. (Ha *et al.*, 2007; Yan *et al.*, 2007). Therefore, treatments using potent inhibitory agents on tyrosinase and melanin formation may be cosmetically useful. Most of the whitening agents (Maeda *et al.*, 1991; Dawley *et al.*, 1993; Nerya *et al.*, 2003) contain hydroxyl (Hong *et al.*, 2008; Lee *et al.*, 2007), aromatic, alkene, carbonyl, and ether groups in their structure, and act as a specific functional group to make the skin white by inhibiting the production of melanin.

During our work on developing potent whiting agents, in order to prevent the inadequacies of current whitening agents (poor skin penetration and toxicity) and maximize the inhibitory effects of melanin creation, we synthesized the title compound, (I), *via* a general chemical reaction, and studied its X-ray crystal structure.

The 3,4-dimethoxyphenyl moiety and 3,4-difluoroaniline group are essentially planar, with a mean deviation of 0.005 and 0.006 Å, respectively, from the corresponding least-squares planes. The dihedral angle between the benzene rings is 53.7 (1)°. The intermolecular N7—H7···O9*^i^* (symmetry code: (*i*) *x*, -*y* + 3/2, *z* - 1/2) hydrogen bond (involving the H atom of the amine and O atom of carbonyl) allows to form an extensive one-dimensional network along the *c*-axis, which stabilizes the crystal structure.

### Experimental

3,4-Difluoroaniline and 3,4-dimethoxy phenyl acetyl chloride were purchased from Sigma Chemicals Co. Solvents used for organic synthesis were distilled before use. All other chemicals and solvents were of analytical grade and used without further purification. The title compound was prepared from the reaction of 3,4-difluoroaniline (1 mmol) and 3,4-dimethoxy phenyl acetyl chloride (1.2 mmol) by simple substitution (nucleophilic addition-elimination on carbonyl C atom) in THF. Removal of solvent gave a white solid. The solid was purified by column chromatography on silica gel (2:1 hexane/ethyl acetate) to give the title compound (92% yield). Colourless crystals (m.p. 393 K) were obtained by slow evaporation of an ethyl acetate solution at 298 K.

### Refinement

Although diffraction data were collected using optimized parameters, a poor quality pattern resulted, which is reflected in the high final residuals. Atom H7 of the NH group was located in a differnce map and refined freely. Other H atoms were positioned geometrically and refined using a riding model, with C—H = 0.93–0.97 Å, and with *U*_iso_(H) = 1.2*U*_eq_(carrier C) for aromatic and CH_2_ groups, and 1.5*U*_eq_(carrier C) for methyl H atoms.

### Crystal data

**Table:**

C_16_H_15_F_2_NO_3_	*F*_000_ = 640
*M**_r_* = 307.29	*D*_x_ = 1.414 Mg m^−^^3^
Monoclinic, *P*2_1_/*c*	Melting point: 393 K
Hall symbol: -P 2ybc	Mo *K*α radiation λ = 0.71073 Å
*a* = 8.6440 (11) Å	Cell parameters from 25 reflections
*b* = 18.867 (6) Å	θ = 10.0–13.5º
*c* = 9.4827 (13) Å	µ = 0.12 mm^−^^1^
β = 111.019 (11)º	*T* = 295 (2) K
*V* = 1443.6 (5) Å^3^	Block, colourless
*Z* = 4	0.26 × 0.26 × 0.23 mm

### Data collection

**Table:**

Enraf–Nonius CAD-4 diffractometer	θ_min_ = 2.2º
non–profiled ω/2θ scans	*h* = −10→9
Absorption correction: none	*k* = 0→22
2855 measured reflections	*l* = 0→11
2689 independent reflections	3 standard reflections
1089 reflections with *I* > 2σ(*I*)	every 400 reflections
*R*_int_ = 0.050	intensity decay: 3%
θ_max_ = 25.5º	

### Refinement

**Table:**

Refinement on *F*^2^	H atoms treated by a mixture of independent and constrained refinement
Least-squares matrix: full	*w* = 1/[σ^2^(*F*_o_^2^) + (0.0403*P*)^2^] where *P* = (*F*_o_^2^ + 2*F*_c_^2^)/3
*R*[*F*^2^ > 2σ(*F*^2^)] = 0.076	(Δ/σ)_max_ < 0.001
*wR*(*F*^2^) = 0.161	Δρ_max_ = 0.19 e Å^−^^3^
*S* = 1.00	Δρ_min_ = −0.19 e Å^−^^3^
2689 reflections	Extinction correction: none
203 parameters	

### Fractional atomic coordinates and isotropic or equivalent isotropic displacement parameters (Å^2^)

**Table:**

	*x*	*y*	*z*	*U*_iso_*/*U*_eq_	
C1	0.1632 (6)	0.6519 (2)	−0.2444 (5)	0.0423 (12)	
C2	0.0544 (6)	0.6516 (3)	−0.3939 (5)	0.0492 (13)	
H2	0.0578	0.6871	−0.4606	0.059*	
C3	−0.0570 (7)	0.5976 (3)	−0.4396 (6)	0.0581 (15)	
C4	−0.0637 (7)	0.5447 (3)	−0.3452 (6)	0.0593 (15)	
C5	0.0438 (7)	0.5443 (3)	−0.1995 (6)	0.0671 (17)	
H5	0.0407	0.5079	−0.1346	0.081*	
C6	0.1575 (6)	0.5985 (3)	−0.1487 (5)	0.0531 (14)	
H6	0.2306	0.5988	−0.0489	0.064*	
F1	−0.1663 (4)	0.59760 (17)	−0.5842 (3)	0.0941 (12)	
F2	−0.1777 (4)	0.49215 (17)	−0.3968 (4)	0.0917 (12)	
N7	0.2760 (5)	0.7098 (2)	−0.2001 (4)	0.0446 (11)	
H7	0.304 (6)	0.729 (3)	−0.273 (5)	0.08 (2)*	
C8	0.3307 (6)	0.7408 (3)	−0.0639 (5)	0.0418 (12)	
O9	0.2978 (4)	0.71902 (17)	0.0431 (3)	0.0608 (11)	
C10	0.4341 (6)	0.8066 (3)	−0.0549 (5)	0.0566 (15)	
H10A	0.5055	0.7986	−0.1122	0.068*	
H10B	0.3607	0.8457	−0.1019	0.068*	
C11	0.5399 (6)	0.8276 (3)	0.1044 (5)	0.0464 (13)	
C12	0.5239 (6)	0.8935 (3)	0.1612 (5)	0.0502 (14)	
H12	0.4463	0.9254	0.1007	0.06*	
C13	0.6219 (6)	0.9127 (3)	0.3069 (5)	0.0490 (14)	
C14	0.7395 (6)	0.8654 (3)	0.3968 (5)	0.0498 (13)	
C15	0.7552 (6)	0.8002 (3)	0.3398 (5)	0.0611 (16)	
H15	0.8334	0.7682	0.3993	0.073*	
C16	0.6557 (6)	0.7815 (3)	0.1945 (6)	0.0599 (15)	
H16	0.6677	0.737	0.1575	0.072*	
O17	0.6123 (4)	0.97621 (19)	0.3725 (4)	0.0741 (12)	
C18	0.4880 (7)	1.0251 (3)	0.2884 (6)	0.0797 (19)	
H18A	0.4946	1.0671	0.3474	0.12*	
H18B	0.3807	1.0039	0.2643	0.12*	
H18C	0.505	1.0373	0.1968	0.12*	
O19	0.8299 (4)	0.88921 (18)	0.5388 (4)	0.0679 (11)	
C20	0.9656 (6)	0.8464 (3)	0.6292 (5)	0.0686 (17)	
H20A	1.0195	0.869	0.7251	0.103*	
H20B	1.0431	0.8408	0.5786	0.103*	
H20C	0.9252	0.8008	0.6446	0.103*	

### Atomic displacement parameters (Å^2^)

**Table:**

	*U*^11^	*U*^22^	*U*^33^	*U*^12^	*U*^13^	*U*^23^
C1	0.047 (3)	0.043 (3)	0.037 (3)	0.001 (3)	0.015 (2)	−0.006 (2)
C2	0.054 (3)	0.045 (3)	0.039 (3)	0.000 (3)	0.006 (3)	0.006 (2)
C3	0.060 (4)	0.062 (4)	0.040 (3)	0.001 (3)	0.004 (3)	−0.013 (3)
C4	0.061 (4)	0.053 (4)	0.060 (4)	−0.007 (3)	0.018 (3)	−0.010 (3)
C5	0.092 (5)	0.043 (4)	0.066 (4)	−0.010 (3)	0.028 (4)	0.001 (3)
C6	0.067 (4)	0.047 (3)	0.040 (3)	0.002 (3)	0.012 (3)	0.006 (3)
F1	0.087 (3)	0.103 (3)	0.062 (2)	−0.025 (2)	−0.0110 (18)	−0.010 (2)
F2	0.093 (3)	0.073 (2)	0.103 (3)	−0.036 (2)	0.028 (2)	−0.024 (2)
N7	0.047 (3)	0.053 (3)	0.030 (2)	−0.004 (2)	0.010 (2)	−0.003 (2)
C8	0.041 (3)	0.053 (3)	0.029 (3)	−0.002 (3)	0.010 (2)	−0.003 (3)
O9	0.078 (3)	0.073 (3)	0.0360 (19)	−0.027 (2)	0.0258 (18)	−0.0142 (18)
C10	0.056 (3)	0.066 (4)	0.045 (3)	−0.009 (3)	0.015 (3)	−0.003 (3)
C11	0.045 (3)	0.051 (3)	0.041 (3)	−0.004 (3)	0.012 (2)	−0.003 (3)
C12	0.044 (3)	0.056 (4)	0.044 (3)	−0.001 (3)	0.009 (3)	0.004 (3)
C13	0.050 (3)	0.049 (3)	0.041 (3)	0.000 (3)	0.007 (3)	−0.011 (3)
C14	0.041 (3)	0.058 (4)	0.041 (3)	−0.001 (3)	0.004 (2)	−0.006 (3)
C15	0.056 (3)	0.057 (4)	0.054 (3)	0.011 (3)	0.000 (3)	−0.014 (3)
C16	0.060 (4)	0.059 (4)	0.057 (3)	0.000 (3)	0.018 (3)	−0.009 (3)
O17	0.069 (3)	0.062 (3)	0.062 (2)	0.020 (2)	−0.012 (2)	−0.008 (2)
C18	0.074 (4)	0.059 (4)	0.083 (4)	0.016 (3)	0.000 (3)	−0.006 (3)
O19	0.062 (2)	0.070 (3)	0.048 (2)	0.019 (2)	−0.0085 (19)	−0.0103 (19)
C20	0.054 (3)	0.078 (4)	0.051 (3)	0.010 (3)	−0.009 (3)	0.000 (3)

### Geometric parameters (Å, °)

**Table:**

C1—C6	1.368 (6)	C11—C16	1.369 (6)
C1—C2	1.392 (6)	C11—C12	1.381 (6)
C1—N7	1.423 (6)	C12—C13	1.386 (6)
C2—C3	1.362 (6)	C12—H12	0.93
C2—H2	0.93	C13—O17	1.367 (5)
C3—C4	1.356 (7)	C13—C14	1.390 (6)
C3—F1	1.357 (5)	C14—O19	1.368 (5)
C4—F2	1.359 (5)	C14—C15	1.370 (6)
C4—C5	1.361 (6)	C15—C16	1.383 (6)
C5—C6	1.380 (6)	C15—H15	0.93
C5—H5	0.93	C16—H16	0.93
C6—H6	0.93	O17—C18	1.423 (5)
N7—C8	1.340 (5)	C18—H18A	0.96
N7—H7	0.89 (5)	C18—H18B	0.96
C8—O9	1.219 (5)	C18—H18C	0.96
C8—C10	1.515 (6)	O19—C20	1.428 (5)
C10—C11	1.511 (6)	C20—H20A	0.96
C10—H10A	0.97	C20—H20B	0.96
C10—H10B	0.97	C20—H20C	0.96
			
C6—C1—C2	120.0 (5)	C16—C11—C10	120.2 (5)
C6—C1—N7	123.6 (4)	C12—C11—C10	121.0 (4)
C2—C1—N7	116.4 (4)	C11—C12—C13	120.9 (5)
C3—C2—C1	118.0 (5)	C11—C12—H12	119.6
C3—C2—H2	121	C13—C12—H12	119.6
C1—C2—H2	121	O17—C13—C12	124.6 (4)
C4—C3—F1	119.2 (5)	O17—C13—C14	115.7 (4)
C4—C3—C2	122.2 (5)	C12—C13—C14	119.7 (5)
F1—C3—C2	118.5 (5)	O19—C14—C15	125.5 (4)
C3—C4—F2	119.8 (5)	O19—C14—C13	115.3 (4)
C3—C4—C5	119.9 (5)	C15—C14—C13	119.2 (4)
F2—C4—C5	120.4 (5)	C14—C15—C16	120.5 (5)
C4—C5—C6	119.5 (5)	C14—C15—H15	119.7
C4—C5—H5	120.2	C16—C15—H15	119.7
C6—C5—H5	120.2	C11—C16—C15	120.9 (5)
C1—C6—C5	120.3 (5)	C11—C16—H16	119.5
C1—C6—H6	119.9	C15—C16—H16	119.5
C5—C6—H6	119.9	C13—O17—C18	118.1 (4)
C8—N7—C1	125.9 (4)	O17—C18—H18A	109.5
C8—N7—H7	118 (3)	O17—C18—H18B	109.5
C1—N7—H7	116 (3)	H18A—C18—H18B	109.5
O9—C8—N7	123.3 (5)	O17—C18—H18C	109.5
O9—C8—C10	122.6 (4)	H18A—C18—H18C	109.5
N7—C8—C10	114.1 (4)	H18B—C18—H18C	109.5
C11—C10—C8	113.8 (4)	C14—O19—C20	117.5 (4)
C11—C10—H10A	108.8	O19—C20—H20A	109.5
C8—C10—H10A	108.8	O19—C20—H20B	109.5
C11—C10—H10B	108.8	H20A—C20—H20B	109.5
C8—C10—H10B	108.8	O19—C20—H20C	109.5
H10A—C10—H10B	107.7	H20A—C20—H20C	109.5
C16—C11—C12	118.8 (4)	H20B—C20—H20C	109.5
			
C6—C1—C2—C3	0.8 (7)	C8—C10—C11—C16	59.3 (6)
N7—C1—C2—C3	−178.9 (4)	C8—C10—C11—C12	−121.7 (5)
C1—C2—C3—C4	−0.6 (8)	C16—C11—C12—C13	−0.6 (7)
C1—C2—C3—F1	178.5 (4)	C10—C11—C12—C13	−179.5 (4)
F1—C3—C4—F2	0.8 (8)	C11—C12—C13—O17	−178.9 (5)
C2—C3—C4—F2	179.9 (5)	C11—C12—C13—C14	0.8 (7)
F1—C3—C4—C5	−179.3 (5)	O17—C13—C14—O19	−0.2 (7)
C2—C3—C4—C5	−0.2 (9)	C12—C13—C14—O19	−180.0 (4)
C3—C4—C5—C6	0.8 (8)	O17—C13—C14—C15	179.1 (5)
F2—C4—C5—C6	−179.3 (5)	C12—C13—C14—C15	−0.6 (8)
C2—C1—C6—C5	−0.2 (7)	O19—C14—C15—C16	179.4 (5)
N7—C1—C6—C5	179.5 (5)	C13—C14—C15—C16	0.2 (8)
C4—C5—C6—C1	−0.6 (8)	C12—C11—C16—C15	0.1 (8)
C6—C1—N7—C8	−34.1 (7)	C10—C11—C16—C15	179.1 (5)
C2—C1—N7—C8	145.6 (5)	C14—C15—C16—C11	0.1 (8)
C1—N7—C8—O9	5.3 (8)	C12—C13—O17—C18	3.0 (7)
C1—N7—C8—C10	−173.2 (4)	C14—C13—O17—C18	−176.8 (5)
O9—C8—C10—C11	20.3 (7)	C15—C14—O19—C20	8.1 (8)
N7—C8—C10—C11	−161.2 (4)	C13—C14—O19—C20	−172.6 (4)

### Hydrogen-bond geometry (Å, °)

**Table:**

*D*—H···*A*	*D*—H	H···*A*	*D*···*A*	*D*—H···*A*
N7—H7···O9^i^	0.89 (5)	1.98 (5)	2.846 (5)	163 (5)

Symmetry codes: (i) *x*, −*y*+3/2, *z*−1/2.

## References

[bb1] Dawley, R. M. & Flurkey, W. H. (1993). *J. Food Sci.* **58**, 609–610.

[bb2] Enraf–Nonius (1994). *CAD-4 EXPRESS* Enraf–Nonius, Delft, The Netherlands.

[bb3] Farrugia, L. J. (1997). *J. Appl. Cryst.* **30**, 565.

[bb4] Farrugia, L. J. (1999). *J. Appl. Cryst.* **32**, 837–838.

[bb5] Ha, Y. M., Chung, S. W., Song, S. H. & Lee, H. J. (2007). *Biol. Pharm. Bull.* **30**, 1711–1715. 10.1248/bpb.30.1711 17827726

[bb6] Harms, K. & Wocadlo, S. (1995). *XCAD4* University of Marburg, Germany.

[bb7] Hong, W. K., Heo, J. Y., Han, B. H., Sung, C. K. & Kang, S. K. (2008). *Acta Cryst.* E**64**, o49. 10.1107/S1600536807062009 PMC2915007 21200924

[bb8] Lee, C. W., Son, E.-M., Kim, H. S., Xu, P., Batmunkh, T., Lee, B. J. & Koo, K. A. (2007). *Bioorg. Med. Chem. Lett.* **17**, 5462–5464. 10.1016/j.bmcl.2007.07.032 17693086

[bb9] Maeda, K. & Fukuda, M. (1991). *J. Soc. Cosmet. Chem.* **42**, 361–368.

[bb10] Nerya, O., Vaya, J., Musa, R., Izrael, S., Ben-Arie, R. & Tamir, S. (2003). *J. Agric. Food Chem.* **51**, 1201–1207. 10.1021/jf020935u 12590456

[bb11] Sheldrick, G. M. (2008). *Acta Cryst.* A**64**, 112–122. 10.1107/S0108767307043930 18156677

[bb12] Yan, H. L., Lin, T. & Zheng, T. W. (2007). *J. Enzyme Inhib. Med. Chem.* **22**, 433–438. 10.1080/14756360601141562 17847709

